# Immunopathogenesis of SARS-CoV-2-induced pneumonia: lessons from influenza virus infection

**DOI:** 10.1186/s41232-020-00148-1

**Published:** 2020-10-12

**Authors:** Masaaki Miyazawa

**Affiliations:** Department of Immunology, Faculty of Medicine, Kindai University, 377-2 Ohno-Higashi, Osaka-Sayama, Osaka 589-8511 Japan

**Keywords:** SARS-CoV-2, Influenza virus, Pneumonia, Tissue injury, Cytokines, Chemokines, T cells

## Abstract

Factors determining the progression of frequently mild or asymptomatic severe acute respiratory syndrome coronavirus-2 (SARS-CoV-2) infection into life-threatening pneumonia remain poorly understood. Viral and host factors involved in the development of diffuse alveolar damage have been extensively studied in influenza virus infection. Influenza is a self-limited upper respiratory tract infection that causes acute and severe systemic symptoms and its spread to the lungs is limited by CD4^+^ T-cell responses. A vicious cycle of CCL2- and CXCL2-mediated inflammatory monocyte and neutrophil infiltration and activation and resultant massive production of effector molecules including tumor necrosis factor (TNF)-α, nitric oxide, and TNF-related apoptosis-inducing ligand are involved in the pathogenesis of progressive tissue injury. SARS-CoV-2 directly infects alveolar epithelial cells and macrophages and induces foci of pulmonary lesions even in asymptomatic individuals. Mechanisms of tissue injury in SARS-CoV-2-induced pneumonia share some aspects with influenza virus infection, but IL-1β seems to play more important roles along with CCL2 and impaired type I interferon signaling might be associated with delayed virus clearance and disease severity. Further, data indicate that preexisting memory CD8^+^ T cells may play important roles in limiting viral spread in the lungs and prevent progression from mild to severe or critical pneumonia. However, it is also possible that T-cell responses are involved in alveolar interstitial inflammation and perhaps endothelial cell injury, the latter of which is characteristic of SARS-CoV-2-induced pathology.

## Background

SARS coronavirus-2 (SARS-CoV-2) is the causative agent of coronavirus disease 2019 (COVID-19)**.** The current pandemic started in Wuhan, China in late 2019 and has caused more than 33 million confirmed infected cases and nearly 1 million deaths as of September 27, 2020 (worldometer COVID-19 CORONAVIRUS PANDEMIC https://www.worldometers.info/coronavirus/). While most cases of SARS-CoV-2 infection are either asymptomatic or mild vis-à-vis clinical signs and symptoms, people with risk factors including obesity, diabetes mellitus, chronic lung disease, heart or renal failure, or those in immunosuppressive states show higher frequencies of developing life-threatening pneumonia. However, in relatively rare cases, pneumonia can develop in younger individuals or in those without known risk factors. Factors that determine the progression of SARS-CoV-2 infection into overt pneumonia are poorly understood.

For hundreds of years prior to the emergence of highly pathogenic human coronaviruses, influenza viruses have been some of the most contagious human respiratory pathogens affecting approximately 9% of the world’s population annually with 300,000 to 500,000 deaths each year [1]. Viral, host cellular, immunological, and genetic correlates of influenza virus-induced pneumonia development have been studied in detail. In this review, I provide a summary of the immunopathogenesis of influenza virus infection first and then attempt to dissect the mechanisms of pneumonia development in SARS-CoV-2 infection drawing on analyses of influenza-induced lung pathologies.

## Clinical course and pathology of influenza virus infection

Seasonal influenza is a self-limited acute viral infection of the upper respiratory tract and in most cases, pneumonic involvement is not clinically prominent [2, 3]. The transmission of human influenza A virus (IAV) occurs through inhalation of infectious droplets or airborne droplet nuclei, and by indirect contact followed by self-inoculation of upper respiratory mucosa or conjunctiva [2]. Both exhalation and coughs of infected individuals have been shown to contain fine droplets that carry small numbers of infectious viral particles at a low frequency [4, 5]. Aerosol inoculation studies of human volunteers suggest that the average risk of infection by a single cough attack is around 10^−4^ and that of contracting the illness is smaller than 10^−4^ [6]. Human IAV attaches predominantly to ciliated tracheal and bronchial epithelial cells and less abundantly to the bronchiolar epithelium [2]. However, viral attachment and effective replication might be discrete processes. In uncomplicated human influenza, virus replication in vivo has been documented in nasal mucosa; ex vivo studies have shown permissiveness of nasopharyngeal, tonsilar, bronchial, and alveolar epithelial cells [2].

The natural course of IAV infection has been studied in human volunteers possessing no detectable serum antibodies (Abs) reactive to the inoculating virus. After intranasal inoculation of A/Texas/36/91 (H1N1), the average virus titer peaked at 2 days after infection. By post-infection day (PID) 8, the nasal virus was almost undetectable [7]. Symptom scores closely followed changes in nasal viral titers, except that nasal discharge weight peaked on day 3. Similarly, when cell culture-grown A/Wisconsin/67/05 (H3N2) virus was intranasally inoculated, virus titers in nasal wash peaked at 2 days after infection and mean symptom scores closely followed, peaking at PID 3 [8].

Pathologically, tracheal and bronchial biopsies of uncomplicated human IAV infection have demonstrated superficial necrotizing tracheobronchitis progressing downwards the respiratory tract between 1 and 7 days after symptom onset [2]. The epithelial layer shows vacuolation, loss of cilia, and desquamation associated with edema and hyperemia of the lamina propria and relatively limited lymphocyte infiltration. As IAV infection causes cytopathic effect and cell death by apoptosis in vitro [9, 10], the above rapidly developing cytopathology likely reflects the direct cytopathic effect of virus infection. As a viral virulence factor, PB1-F2 encoded by the PB1 segment of the viral RNA genome preferentially localizes to mitochondria and induces apoptosis [11]. In fact, the desquamation of the epithelium in the tracheobronchial tree is multifocal and irregularly distributed [3], conceivably reflecting the distribution of virus-infected cell foci. Neutrophils are absent in the early stage of infection, but they rather migrate when epithelial cell death takes place, followed later by mononuclear cells [3]. Thus, in IAV-induced tracheobronchitis, initial epithelial cell death is caused by the virus infection itself, and neutrophil infiltration is a result of tissue injury. Pathological changes in the bronchial epithelium are short-lasting and often show only in the thickening of the epithelium corresponding to a regenerative process and slight increases in lymphocyte infiltration between 1 and 6 days after symptom onset. In fact, the process of epithelial repair starts as early as 2 days after symptom onset [2].

**Fig. 1:**
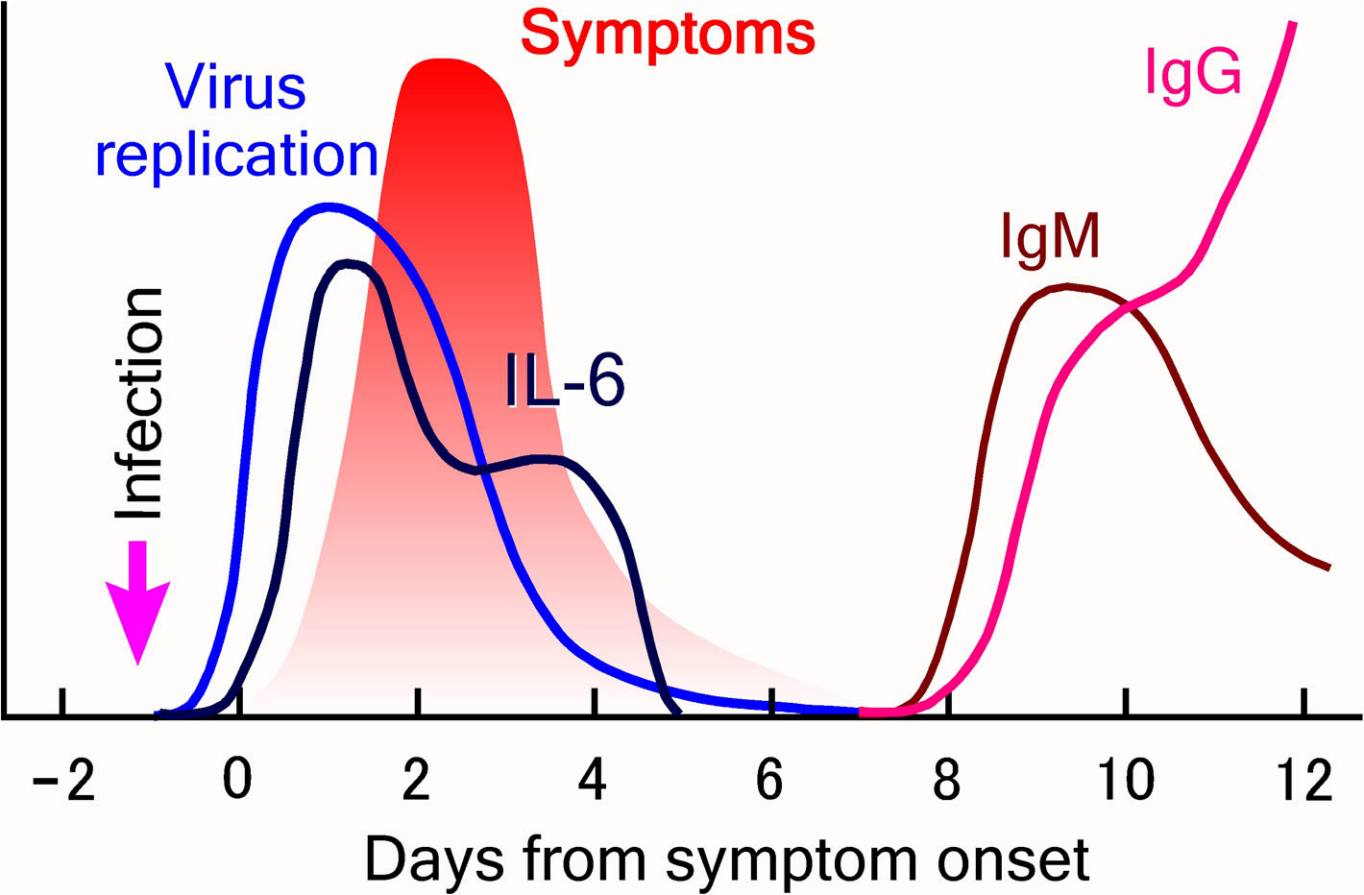
Diagrammatic representation of the kinetics of IAV infection. Time course of virus replication, cytokine production, and Ab responses are summarized based on data from references [7, 8, 12]

Although uncomplicated influenza virus infection is self-limited, it nevertheless causes sudden onset of major systemic symptoms starting with malaise and high fever, followed by respiratory manifestations of coryza and cough often associated with headache, myalgia, and/or arthralgia [3]. These systemic symptoms coincide with local and systemic cytokine responses (Fig. 1). In experimental human infection with A/Texas/36/91 (H1N1) virus, amounts of IL-6 and IFN-α in nasal lavage fluid peaked at 2 days after infection followed by TNF-α and IL-8 peaked at PID 4 [7]. Similarly, serum IL-6 peaked at 2 days after infection. Body temperature and upper respiratory tract symptoms as well as nasal cytokine levels at PID 2 correlate strongly with virus titers, and plasma IL-6 levels at PID 3 showed the strongest correlation with total symptoms. In the cases of 2009 pandemic H1N1 influenza A virus (H1N1pdm) infection, serum IL-6 levels were significantly higher in hospitalized critical versus non-critical cases, and experimental infections of several different strains of mice with A/Mexico/4108/09 (H1N1) virus induced the expression of IL-6 mRNA in their lungs at 3 days after infection [13]. However, the lack of IL-6 in the knock-out strain of mice did not significantly affect survival or lung pathology. Thus, IL-6 produced in the upper respiratory tract seems to be mainly responsible for the induction of systemic symptoms in infected humans, while IL-6 is not directly involved in progression to pneumonia development.

Sources of the proinflammatory cytokines may include epithelial cells of the upper respiratory tract and tissue-resident macrophages [7]. In fact, cultured human bronchial epithelial cells produced significant amounts of IL-6, IL-8, and CCL5 upon infection with A/Shisen/2/93 (H3N2) virus detectable from 24 h after infection [14]. Following the attachment of IAV particles to the plasma membrane through HA targeting of sialic acids, viral particles are endocytosed and the fusion of viral envelope to the vesicular membrane results in the release of viral RNA into the cytoplasm. Endosomal detection of viral RNA species activates Toll-like receptors (TLRs) 3 and 7, while the presence of viral nucleic acids in the cytosol activates RIG-I and NLRP3 inflammasome pathways [11, 15]. These result in the expression of type I interferons (IFNs) and the production and processing of IL-1β and IL-18 in infected epithelial cells. Type I IFNs are classically believed to contribute to the curtailment of epithelial cell infection by rendering uninfected cells resistant to virus spread (reviewed in [11]). Bimodal changes in IFN-α titers in experimentally infected human volunteers [7] have been reproduced in mathematical models by incorporating reductions in the rate of virus replication through the effect of IFN-α [12]. After infection of epithelial cells, tissue-resident macrophages respond to viral infection and phagocytose both viral particles and apoptotic epithelial cells. These resident macrophages as well as tissue-resident intraepithelial and interstitial dendritic cells (DCs) are sources of multiple proinflammatory cytokines including IL-6 [15, 16].

## Spread of infection from the upper to lower respiratory tract

Although macrophages play an important role in the initial clearance of viral particles and infected apoptotic cells, the eventual elimination of virus-producing epithelial cells depends on the activation of effector T cells [16, 17]. In this regard, seasonal influenza usually causes higher mortality in the elderly, while a pandemic of IAV infection often results in disproportionate mortality in younger individuals [3], suggesting that partial immunity conferred by previous exposure to historically circulated virus strains may have protected otherwise susceptible older individuals [15]. In fact, several human studies have shown that pre-existing IAV-specific T-cell responses are associated with reduced virus shedding and/or illness upon natural or experimental infection (reviewed in [18]). In intranasal challenge studies of seronegative healthy volunteers, pre-existing CD4^+^, but not CD8^+^, T cells responding to conserved IAV nucleoprotein or matrix protein epitopes were detectable at 7 days after infection when no serum Abs were detectable against the challenging virus and were significantly associated with reduced viral shedding, symptom scores, and illness duration [8]. In mouse models of IAV infection, adoptive transfer of memory Th1 or Th17 cells induced protection through the direct effect of IFN-γ production or by the activation of multiple downstream effector mechanisms in the recipients [18]. It should be noted that lymphocyte infiltration into the bronchial epithelium is observed between 1 and 6 days post**-**symptom onset upon human IAV infection [2]. Further, in mice experimentally infected with A/Hong Kong/X31 (H3N2) virus, a short-term antiviral IgM response peaked at around PID 10 and IgG responses peaked later at around PID 20, both of which take place some time after the decrease in lung virus titers [19]. Thus, it is plausible that although innate immune responses limit IAV replication in the tracheobronchial epithelium, early T-cell responses are associated with eventual viral clearance and contribute to prevent the spread of infection toward the lower respiratory tract. It should be noted, however, that in macaque infection with A/Kawasaki/173/01 (H1N1) peribronchiolitis with the formation of lymph follicles was observed throughout the lung with no viral antigens detectable at PID 8 [20], suggesting that local Ab production may initiate at least as quickly as CD4^+^ T-cell responses and may contribute to viral antigen clearance prior to the detection of serum Abs.

## Pathology of influenza-induced pneumonia

While seasonal human IAV infection causes temporal, transient tracheobronchitis, the extension of viral infection to alveoli can result in severe pneumonia frequently associated with concomitant or secondary bacterial pneumonia [2, 3]. Whether the otherwise self-limited tracheobronchitis progresses to potentially fatal pneumonia or not is thought to be determined by both viral and host factors [11]. The HA of seasonal IAV binds to α_2-6_ sialylated glycans that are mainly expressed on the epithelial cells of the upper respiratory tract in humans. On the other hand, the HA of highly pathogenic avian H5N1 viruses preferentially binds to α_2-3_ sialylated glycans and strongly attaches to type II pneumocytes and alveolar macrophages, resultantly causing severe pneumonia [2, 11, 15]. However, as primary target cells of infection are located deep in the alveoli, human-to-human transmission of avian viruses is rare. These differences in tissue tropism of the viruses partly explain different pathogenicities of IAV subtypes. Of particular interest, the H1N1pdm virus that caused the 2009 pandemic is adapted to bind to both α_2-6_- and α_2-3_-linked sialylated glycans [7, 11, 21], and the levels of pulmonary replication of this IAV subtype is higher than those of seasonal IAV [7, 21]. Host-related risk factors for the development of pneumonia include the lack of previous exposure to the infecting subtype, older age, and background medical conditions, all associated with diminished immune responses [2]. Protection of the lungs from IAV infection is at least partly mediated by IgG Abs specific for viral surface glycoproteins. Serum IgG supposedly reaches the alveolar lining through transudation from the blood vessels [2]. However, it has been shown that in the presence of IAV-specific memory CD4^+^ T cells, rapid elimination of the virus and reduced morbidity can be observed in the absence of Ab production [8, 18].

The prototypic pathology of fatal IAV-induced pneumonia in pandemic cases is diffuse alveolar damage (DAD) that is characterized by denudation of alveolar septa and desquamation of epithelial cells into the lumen [2, 3, 21]. Desquamated alveolar epithelial cells show pyknosis and karyorrhexis indicating apoptotic death. Both type I and type II alveolar epithelial cells are damaged. As type I cells comprise the alveolar-capillary leakage barrier and type II cells resorb fluid from alveolar lumina, damage to these two types of pneumocytes along with an active increase in vascular permeability under inflammatory stimuli to endothelial cells [22, 23] cause flooding of alveolar lumina. The above fluid leakage and intra-alveolar hemorrhage, along with the lack of resorption cause hyaline membrane formation. Cellular infiltrates into widened alveolar septa are mainly neutrophils and a few eosinophils. In the late stage, type II pneumocyte hyperplasia and metaplasia shows regenerative changes with septal fibrosis and lymphocyte infiltration [2, 3].

The pathology of interpandemic IAV pneumonia is essentially the same as that of pandemic cases. Interestingly, pathological studies of interpandemic cases showed the presence of viral antigens in tracheobronchial and bronchiolar epithelial cells, but not in the alveolar epithelium or macrophages even in cases of DAD [2]. These observations indicate that DAD can develop independently of IAV replication in alveoli.

## Mechanisms of tissue injury in influenza-induced pneumonia

As discussed above, cross-reactive memory CD4^+^ T cells can block the spread of IAV infection from the upper to lower respiratory tract by functioning as a first-line of defense to newly emerged or variant subtypes that evade neutralizing Ab responses [8, 24]. However, recall responses of memory T cells may also promote tissue injury due to differences from primary responses of naive T cells in respect of function, kinetics, and spatial distribution. In fact, while pre-existing memory CD4^+^ T cells conferred partial protection of mice from lethal IAV challenge, the blockade of costimulatory signaling with CTLA4-Ig treatment of the memory-possessing mice even resulted in improved survival [25]. Severe consolidation around bronchial trees observed in IAV-infected mice possessing memory CD4^+^ T cells was ameliorated by CTLA4-Ig treatment, indicating that the formation of lung consolidation is at least partly mediated by sensitized CD4^+^ T cells.

**Fig. 2:**
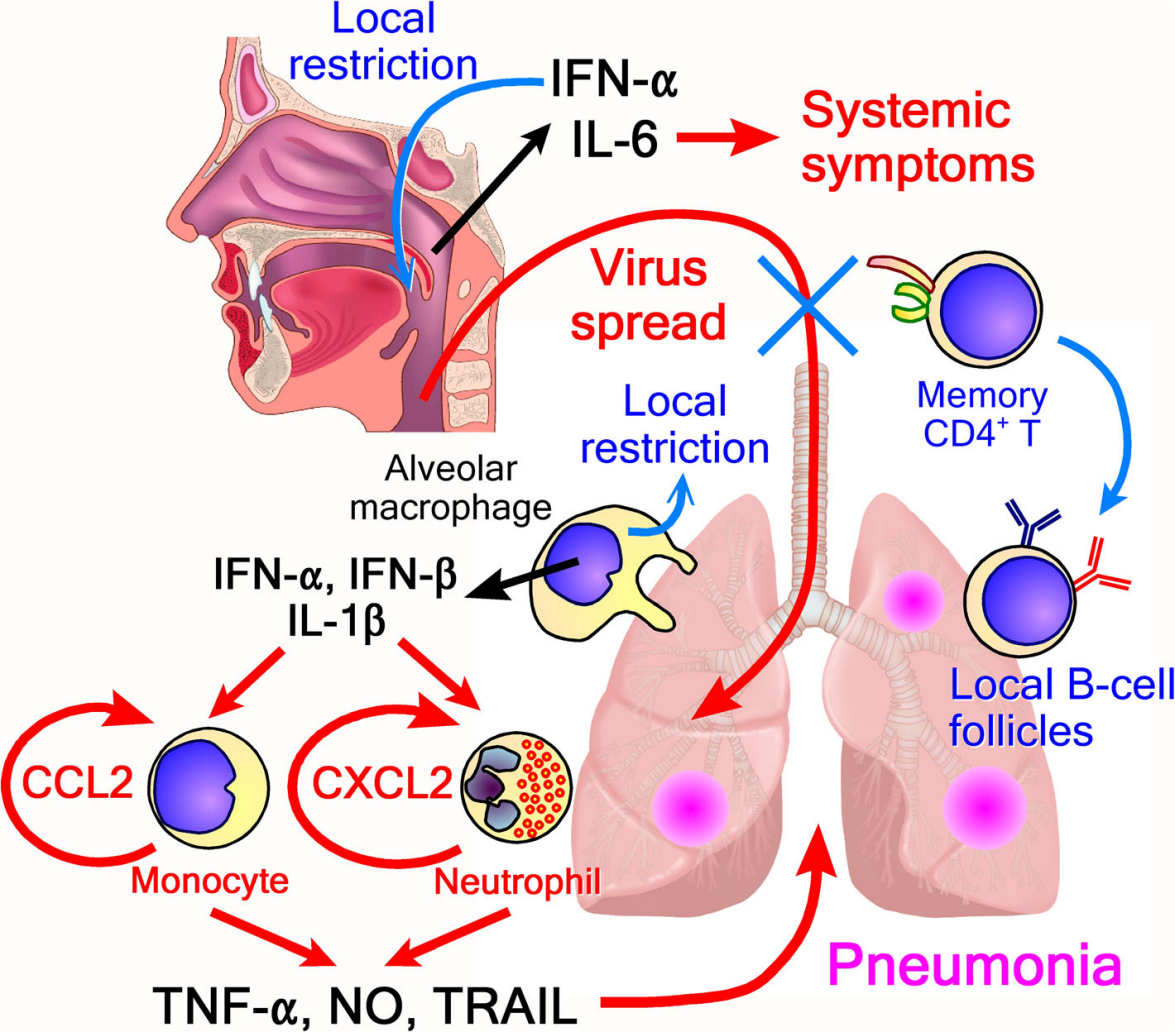
Schematic representation of the pathogenesis of IAV-induced lung injury. See the main text for a detailed explanation

Although the contribution of memory CD4^+^ T cells in inducing lung pathology has been clearly shown in mouse models, in histopathological analyses of human IAV-induced pneumonia lymphocyte infiltration was observed only in later stages [2, 3], indicating that innate immune responses must play more important roles in the early phase of lung injury (Fig. 2). In fact, upon IAV infection in the lungs, tissue-resident alveolar macrophages are the first responders to IAV and serve as a primary source of type I IFNs. In a mouse model of pandemic IAV infection in which a recombinant virus expressing the HA and NA from the 1918 pandemic H1N1 virus in the genetic background of A/Texas/36/91 (H1N1) was used [26], depletion of alveolar macrophages prior to sublethal infection resulted in uncontrolled virus replication and 100% mortality associated with significantly reduced IFN-α and IFN-γ production, indicating that resident alveolar macrophages were functioning to promote viral clearance and thereby limited tissue injury. Interestingly, the 2009 pandemic H1N1 virus, unlike seasonal IAVs, has been reported not to activate innate antiviral responses in human DCs and macrophages despite similar levels of inflammatory mediator responses [21]. However, the release of proinflammatory cytokines and chemokines from infected epithelial cells and activated resident alveolar macrophages induce the recruitment and differentiation of monocytes and DC precursors along with neutrophils. Monocyte-derived macrophages produce higher levels of proinflammatory cytokines and effector molecules including TNF-α and inducible nitric oxide synthetase (NOS) than resident alveolar macrophages and these can promote alveolar injury [15]. In fact, in a mouse model of A/Puerto Rico/8/34 (H1N1) virus infection, a peak of type I IFN production into bronchoalveolar lavage fluid (BALF) was observed at PID 3 and neutralization of either IFN-α or IFN-β significantly reduced alveolar epithelial cell apoptosis at PID 8 [27]. It has been shown that the pro-apoptotic TNF-related apoptosis-inducing ligand (TRAIL) is upregulated in alveolar macrophages of patients with IAV-induced acute respiratory distress syndrome (ARDS) in comparison with those suffering non-viral ARDS [27]. Further, macrophage TRAIL expression is rapidly upregulated upon IFN-β treatment.

Upon infection of wild-type (WT) mice with A/Puerto Rico/8/34 (H1N1) virus, the vast majority of inflammatory cells present in the lungs by PID 5 were derived from CCR2^+^ inflammatory monocytes [28]. Lung numbers of monocytes and monocyte-derived DCs, but not of resident macrophages, were reduced in CCR2-deficient mice along with NOS2-expressing cells. These reductions in inflammatory cells in CCR2-deficient mice were associated with markedly reduced protein content in BALF, morbidity, and mortality without affecting lung virus titers [28]. Thus, these results collectively indicate that resident alveolar macrophages contribute to clear lung viruses, while IAV-induced alveolar injury is primarily mediated by monocyte-derived cells through the production of TNF-α and NO and upregulation of TRAIL [29].

Finally, systematic flow cytometric, gene expression, and tissue imaging comparisons between lethal and sublethal IAV infections of mice revealed that infectious spread, not virus titers of the entire lung, clearly distinguish lethal from sublethal infection [30]. At the same infectious particle load, the more highly pathogenic strain of IAV spread much more widely through the lung tissues than a less pathogenic one. Poorly controlled infectious spread during the early innate stage lead to the activation of self-reflexive feed-forward loops of chemokines which resulted in lung accumulation of neutrophils and monocytes. Activated neutrophils produced neutrophil chemokine CXCL2 and expressed TNF-α and IL-1α in addition to very high levels of pro-IL-1β, and these proinflammatory neutrophils were major correlates of lethal infection. Lung infiltrating monocytes produced CCL2. Thus, in the process of lethal IAV infection, neutrophils and monocytes amplify their own recruitment and induce tissue injury (Fig. 2). These findings are consistent with those obtained from the experimental lethal infection of macaques [20]: when the spread of the virus is confined in small foci, viral antigen-positive cells can be eliminated by PID 8 with resultant formation of peribronchiolar lymphoid follicles. On the other hand, when the infection is more widespread spatially, peaks of CCL2 and IL-8 in sera were observed on day 6 of infection followed later by a peak of serum IL-6 on day 8, and DAD lesions were observed in areas surrounding those of virus-antigen positive cells at PID 8. Therefore, excessive production of proinflammatory cytokines and chemokines induces a vicious cycle of monocyte and neutrophil recruitment and activation, resultantly causing DAD even in areas surrounding actual foci of virus replication.

## Replication of SARS-CoV-2 and clinical course of its infection

The cellular process of SARS-CoV-2 infection starts with the attachment of viral particles to target cell plasma membrane through the binding of surface spike (S) glycoprotein with its cellular receptor angiotensin-converting enzyme 2 (ACE2) [31–33]. Following endocytosis of attached virions, acid-dependent proteolytic cleavage of S glycoprotein by cellular protease TMPRSS2 activates the process of fusion between the viral envelope and cellular membrane [34, 35]. Immunohistochemical examinations have demonstrated the presence of ACE2 protein in endothelial and smooth muscle cells of arterioles and large arteries in all examined tissues. ACE2 is highly expressed in both type I and type II pneumocytes as well as brush borders of small intestinal epithelial cells, and weakly in bronchial epithelial cells and basal layers of oral and nasopharyngeal stratified epithelial tissues [36]. Single-cell transcriptomic analyses of healthy human tissues have revealed that nasal goblet cells and ciliated epithelial cells display the highest expression levels of the *ACE2* gene within the respiratory system, and TMPRSS2 is expressed in a subset of ACE2-positive cells [37]. A separate meta-analysis of human, non-human primate, and mouse single-cell RNA-seq datasets [38] has shown that the *ACE2* and *TMPRSS2* genes are selectively co-expressed in small percentages of type II pneumocytes both in healthy adult macaques and in fibrotic human lungs, and in a higher percentage of ciliated epithelial cells in the latter. Further, *ACE2* gene expression in primary bronchial cells was substantially upregulated with type I IFN treatment [38]. These patterns of ACE2 expression are consistent with the fact that SARS-CoV-2 as well as the severe acute respiratory syndrome coronavirus (SARS-CoV) can initially cause lower respiratory tract diseases [39].

Unlike IAV that replicates and transcribes its negative-sense RNA genome within the nucleus of infected cells [15], coronaviruses replicate and transcribe their positive-sense RNA genome within the host cell cytoplasm [34, 39]. Nearly two-thirds of the coronavirus genome is dedicated to encode two partly overlapping open-reading frames (ORFs), ORF1a and 1b, the products of which are the largest known RNA virus polyproteins [39]. The above co-terminal polyproteins are proteolytically processed to a large number of non-structural proteins (NSPs) and assemble, together with recruited host cell proteins, to form the replication and transcription complexes (RTCs) located to a network of perinuclear membrane structures [39]. Many of NSPs are multifunctional, and some contribute to enhance viral replication by targeting host gene expression and innate cellular defense mechanisms [34, 39].

**Fig. 3:**
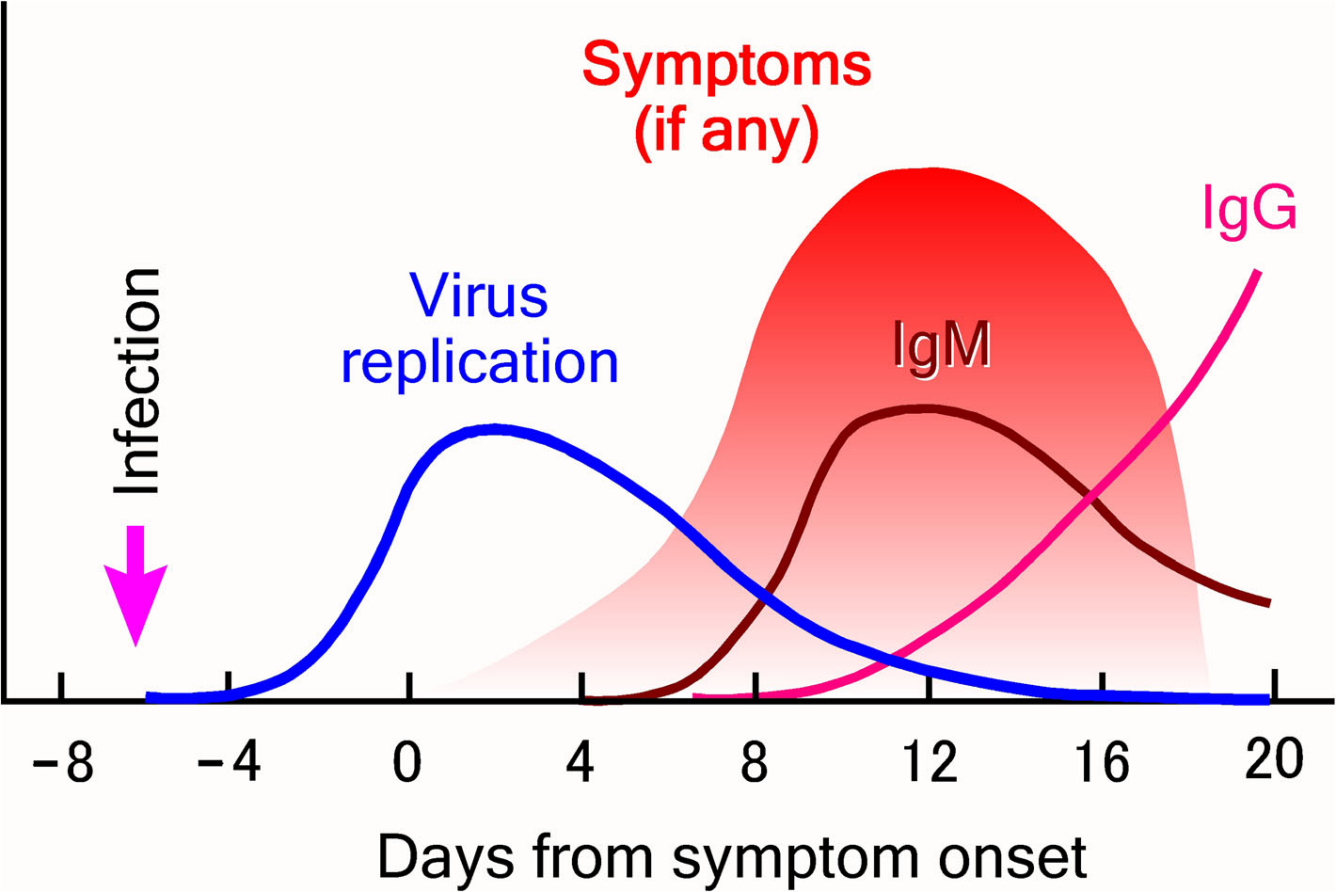
Diagrammatic representation of the kinetics of SARS-CoV-2 infection. Time course of Ab responses is based on reference [40]

Upon infection with positive-sense RNA viruses including coronaviruses, innate cellular responses are triggered by the double-stranded and 5’-triphosphated RNA species that are recognized by RIG-I and MDA-5 in most host cell types (reviewed in [39]). TLR3 is also known to recognize coronaviruses. Thus, type I IFN and proinflammatory cytokines including IL-1β are produced from coronavirus-infected cells. A recent genetic analysis of young cases of severe COVID-19 [41] has revealed decreased type I and type II IFN responses in the absence of TLR7 expression due to a 4-nucleotide deletion or a missense mutation within the X-chromosomal *TLR7* gene. Although TLR7 stimulation with SARS-CoV-2 was not directly examined, this study suggests that TLR7-mediated IFN responses might protect against COVID-19 progression. Coronaviruses, however, employ elaborate mechanisms, including the above formation of replication organelles, to hide their viral replication machinery from the cytosolic innate sensors. For example, besides inhibiting cellular mRNA translation, nsp1 of SARS-CoV is shown to block type I IFN signaling in infected cells by reducing phosphorylated STAT1 [39]. Further, in addition to the ORFs encoding the replicase subunits that are conserved among all coronaviruses, several less conserved downstream ORFs encode accessory proteins that may function to suppress innate immune responses. SARS-CoV ORF3b and ORF6 are known to block IFN production and/or signaling (reviewed in [39]). SARS-CoV-2 ORF3b shows even higher activity than corresponding SARS-CoV protein in antagonizing IFN-β1 promoter function due to the presence of premature termination codons in the *ORF3b* gene [42]. Therefore, unlike IAV infection that causes severe systemic symptoms soon after infection, SARS-CoV-2 infection progresses more slowly than IAV infection and induces rather mild or even unnoticeable local and systemic symptoms in the initial phase of infection (Fig. 3).

As to virological and clinical kinetics of human SARS-CoV-2 infection, exposure history and illness timelines of laboratory-confirmed cases, including information pertaining to cluster cases, have revealed the distribution of days from infection to symptom onset with the mean incubation period of 5.2 days [43]. Virus titers in throat swabs were highest on the day of symptom onset and declined thereafter. Infectiousness is estimated to start from 2.3 days before symptom onset and decline quickly within 7 days [44]. In the assessment of RT-PCR-confirmed asymptomatic SARS-CoV-2 infection [45], the median duration of virus shedding was 19 days, which was significantly longer than that of symptomatic cases. These data are diagrammatically summarized in Fig. 3. At approximately 3–4 weeks after exposure, virus-specific serum IgG titers were significantly lower in asymptomatic than in symptomatic cases, and IgG and virus-neutralizing Ab titers declined both in asymptomatic and symptomatic cases between acute and convalescent phases. Serum IL-6, IL-2, IL-15, the monocyte chemokine CCL2, as well as TRAIL levels were significantly higher in symptomatic than in asymptomatic cases, indicating a possible association of these cytokines and chemokines with disease pathogenesis. Interestingly, chest CT examinations demonstrated small foci of ground-glass opacity in the lung in 29.7% of asymptomatic individuals. Similar CT findings of lung opacities are reported for 54% of RT-PCR-positive asymptomatic cases from the cruise ship docked at Yokohama Bay, Japan [46], indicating that lung lesions are present even in the absence of clinical symptoms.

## Pathology of SARS-CoV-2-induced pneumonia

SARS-CoV-2 infection in humans can induce diverse, multi-organ pathologies in the lungs, heart, kidneys, central nervous system, hematopoietic and lymphoid tissues, and vasculature [47]. Histopathological examinations of autopsy and biopsy cases of COVID-19 revealed features closely resemble those found in SARS and Middle East Respiratory Syndrome (MERS) cases. Post-mortem findings in the lungs of RT-PCR-confirmed COVID-19 patients with a mean time from symptom onset to death of 16 days [48] revealed features of exudative and early and intermediate proliferative phases of DAD. The early exudative changes of DAD including capillary congestion, interstitial and intra-alveolar edema, and hyaline membrane formation were observed in all cases with platelet-fibrin thrombi in small arterioles found in 87% of them. Type II pneumocyte hyperplasia characterizing early proliferative phase was observed to some extent in all cases with intraalveolar granulation in about half of the cases. Importantly, all cases showed radiographic features of interstitial pneumonia at the time of hospitalization, and foci of lymphocyte infiltration along the thickened interalveolar septa were also observed in post-mortem lung specimens. The inflammatory components were CD3^+^ T cells infiltrating into the interstitium and macrophages in alveolar lumina [48]. Similar findings of exudative and early proliferative phases of DAD with interstitial mononuclear cell infiltration dominated by lymphocytes were also reported in another series of autopsy cases [49]. Interestingly, in a significant proportion of autopsy cases, epicardial mononuclear cell infiltration with the predominance of CD4^+^ T cells was observed [47]. Hemophagocytosis indicative of excessive cytokine-induced macrophage activation was also observed in the bone marrow and the spleen in significant numbers of cases.

Most histopathological findings in the lungs of COVID-19 cases are common to viral pneumonia in general, but COVIOD-19-induced DAD is characterized by damages to alveolar capillary endothelium and type II pneumocytes, leading not only to alveolar septal edema and hyaline membrane formation but also to accumulation and aggregation of platelets and megakaryocytes in alveolar capillary lumina and precipitation of fibrin [50]. Although the above pulmonary thrombotic microangiopathy can be observed in DAD of other etiologies including IAV, the thrombotic changes observed in COVID-19 DAD are severer and more extensive. In this regard, SARS-CoV-2 can directly infect endothelial cells and lymphocytic endotheliitis in multiple organs were histopathologically observed in patients’ specimens [51].

Although autopsy cases have provided valuable information on the pathophysiology of the advanced or end-stage disease, the pathogenesis of initial tissue injury might not be reflected in the advanced lesions. In this regard, pathological changes in the lungs resected for cancer that were retrospectively found to be positive with SARS-CoV-2 infection have been reported [52]. Among these, one case was non-fatal and the patient gradually recovered from bilateral viral pneumonia developed after lobectomy. The resected lung showed patchy foci of proteinaceous exudate with thickening of alveolar walls with mononuclear cell infiltration and type II pneumocyte hyperplasia. Some areas showed abundant alveolar macrophages along with pneumocyte hyperplasia. Thus, pathological changes in non-fatal lung lesions would be similar to those observed in fatal cases except less extensive spatial distribution in the former.

**Fig. 4:**
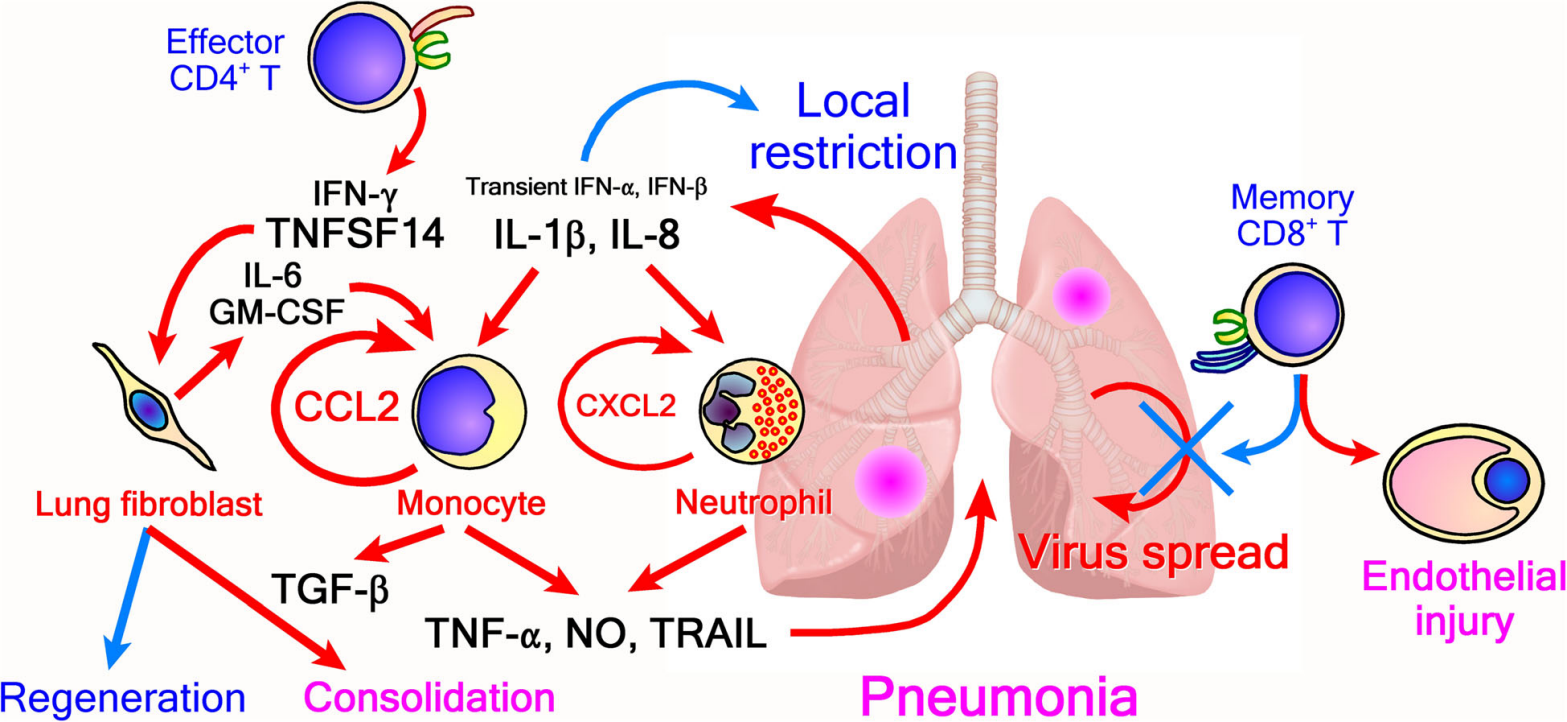
Schematic representation of the pathogenesis of SARS-CoV-2-induced lung injury. See the main text for a detailed explanation

Natural courses of SARS-CoV-2 infection have also been analyzed in non-human primate models. In adult macaques experimentally inoculated intranasally and intratracheally with a SARS-CoV-2 isolate [53], body temperature spiked on PID 1 but returned to normal. Clinical scores of symptoms peaked on PID 4 followed by bodyweight reduction peaking around 7 to 10 days after inoculation. Neutropenia started to be observed on PID 3 and persisted for 2 weeks. Viral load in nose and throat swabs were highest on PID 1 and declined thereafter, probably reflecting a lack of vigorous virus replication in the nasopharyngeal and tracheal mucosa. Nevertheless, a spike of IL-6, IL-15, CCL2, and CCL4 was observed on PID 1, and anti-S glycoprotein and neutralizing Abs started to appear in the serum by PID 10. Radiographic patches of pulmonary infiltration started to appear on PID 1 and progressed to extend and spread through PID 3 and 5, and disappeared by PID 14. Histopathological lung consolidations were observed in animals euthanized at PID 3 and were characterized by massive mononuclear cell infiltration. Viral antigen was detected sporadically in nasal and tracheal epithelial layers and in tonsillar macrophages. These sporadic distributions of viral antigens in the upper respiratory tract are consistent with reported patterns of ACE2 and TMPRSS2 coexpression [37, 38]. Ultrastructural examination of lungs revealed virus shedding from type 2 pneumocytes with interstitial mononuclear cell infiltration. It should be pointed out that one electron micrograph clearly shows the budding of viral particles from endothelial cells possessing characteristic transcytotic vesicles.

In a separate experiment in which older rhesus macaques were inoculated with a higher dose of SARS-CoV-2 [54], viral loads in oropharyngeal and nasal swabs showed a peak at around 5 days after an intratracheal inoculation indicating successful replication of the virus, although no significant changes in body temperature and weights were observed. Viral loads in the bronchi and lungs were detectable and increased between PID 3 and 6, and patches of ground-glass opacity were detected in the lungs starting from PID 1. Pathological findings include massive consolidation of the lungs with thickened alveolar walls and significant monocyte infiltration, hyaline membrane formation, and hemorrhage. These observations indicate again that unlike seasonal IAV which replicate first in the upper respiratory tract and can spread downward to the lower part and may cause pneumonia, SARS-CoV-2 can directly infect the lower respiratory tract and rapidly induce alveolar lesions without causing much systemic symptoms.

SARS-CoV-2 infection of human ACE2-transgenic mice in which ACE2 expression is driven by the cytokeratin-18 promoter [55] also revealed a progressive inflammatory process starting at PID 2 in perivascular sites. By PID 7, massive neutrophil and monocyte infiltration has extended throughout the lungs causing consolidation. Interestingly, viral RNA expression was diffusely spread through the lungs on PID 4 but was markedly diminished and confined in collapsed alveoli by PID 7, indicating that the consolidating lung pathology was caused by host responses following virus replication.

## Immunopathogenesis of SARS-CoV-2-induced pneumonia

As in the case of IAV infection, the development of severe lung pathology upon SARS-CoV-2 infection does not seem to be solely determined by viral loads [56]. In a study of SARS-CoV infection in mice, abrogation of type I IFN signaling by utilizing the *Ifnar*^*−/−*^ strain of mice deficient of IFNαβ receptor or by the administration of an anti-IFNα receptor Ab resulted in reduced morbidity and mortality without significant changes in viral loads. Alveolar edema and thickening of the interstitium observed at PID 5 in untreated WT mice were markedly reduced in *Ifnar*^*−/−*^ mice. Despite that lung virus titers and distribution and numbers of virus antigen-positive cells were not different between untreated WT and *Ifnar*^*−/−*^ animals, significant induction of IFN-β and CCL2 that peaked at 48 h after infection in untreated WT mice were not observed, and infiltration of CCR2^+^ inflammatory monocyte-derived macrophages peaking at PID 3 in WT mice was drastically reduced in mice lacking type I IFN signaling. Further, Ab-induced depletion of inflammatory monocytes resulted in protection from lethal infection and amelioration of pulmonary pathology in young WT animals. In addition, neutralization of IFN-α also ameliorated morbidity and mortality in SARS-CoV-infected WT mice [57]. In a mouse model of MERS coronavirus (MERS-CoV) infection, type I IFN administration within 1 day after infection resulted in protection from lethal challenge, while delayed IFN-β treatment on PID 2 and 4 caused a significantly elevated expression of CCL2, increased infiltration of monocytes and neutrophils into the lungs, and development of fatal pneumonia in sublethally infected animals [58]. Importantly, the administration of anti-CCR2 Ab to MERS-CoV-infected and IFN-β-treated animals improved their survival.

These experimental data indicate that similar to the above-described IAV-induced pneumonia induction, the production of type I IFNs in infected lung tissue can result in excessive infiltration of monocytes and neutrophils and CCL2-mediated accumulation of monocyte-derived macrophages are at least partly responsible for the generation of tissue injury in SARS-CoV infection (Fig. 4). It should be noted, however, that in ex vivo comparisons of SARS-CoV-2 and SARS-CoV infection in human lung tissues, although SARS-CoV-2 infected both type I and type II pneumocytes and alveolar macrophages and replicated more efficiently than SARS-CoV, SASR-CoV infection resulted in higher levels of type I, type II, and type III IFN responses than SASR-CoV-2 infection [59]. The above-reduced production of type I IFNs upon SARS-CoV-2 infection in comparison with SARS-CoV infection may be associated with the potent IFN antagonist activity of SARS-CoV-2 ORF3b [42]. Instead, significant levels of IL-6 and IL-1β responses were observed at 24 and 48 h after infection with SARS-CoV-2, respectively. Similarly, CXCL1 and CXCL5 were significantly induced in the human lungs at 24 h after infection with SARS-CoV-2. Thus, in the case of human SARS-CoV-2 infection, IL-1β might play more important roles than type I IFNs in inducing inflammatory tissue injury in the lungs.

Metatranscriptome sequencing analyses revealed that proinflammatory cytokine and chemokine genes including those encoding IL-1β, DC/monocyte attractant CXCL17, CXCL8 (IL-8), and CCL2 were upregulated in BALF samples obtained from COVID-19 patients in comparison with those from healthy control individuals [60]. Global functional analyses indicated the upregulation of IFN signaling pathways in association with SARS-CoV-2 infection. Composition analyses showed higher neutrophil counts in SARS-CoV-2 samples. Most importantly, single-cell RNA-seq analyses of BALF cells from a relatively small number of patients with varying severity of COVID-19 [61] revealed that BALFs from severe/critical COVID-19 patients contains significantly higher proportions of macrophages and neutrophils but lower proportions of myeloid and plasmacytoid DCs and T cells than those from moderate cases. In accordance with other studies, BALFs from patients with severe/critical infection showed much higher levels of IL-8, IL-6, and IL-1β expression than those from patients with moderate disease. Further, it was suggested that a highly proinflammatory macrophage microenvironment is present in the lungs of severe CIVID-19 patents. Interestingly, single-cell T-cell receptor sequencing showed that CD8^+^ T-cell numbers and their levels of clonal expansion differ between patients with moderate versus severe/critical illness. Thus, CD8^+^ T cells in patients with moderate infection showed limited T-cell receptor repertoire and higher clonal amplification while those in severe/critical patients showed higher proliferating and CCR7^+^ proportions. Tissue-resident signature score of CD8^+^ T cells was higher in moderate cases than in severe ones. Therefore, pre-existing lung-resident memory CD8^+^ T cells may enable better control of SRS-CoV-2 infection in the lungs [56]. Indeed, significant CD4^+^ T-cell responses to SARS-CoV-2 spike and non-spike epitopes have been detected in 40–60% of unexposed individuals [62], suggesting the presence of pre-existing T cells cross-reactive between endemic, common-cold human coronaviruses and SARS-VoV-2. Similarly, SASR-CoV-2 S glycoprotein-reactive CD4^+^ T cells were detected in the peripheral blood of 83% of COVID-19 patients but also in 35% of healthy donors [63]. Interestingly, CD4^+^ T cells from healthy donors reacted primarily to C-terminal epitopes conserved among human coronaviruses.

The above data on BALF samples are largely consistent with those of systems biological assessments which compared mild/moderate and severe/critical COVID-19 cases using blood samples [64, 65]. Thus, CD4^+^ and CD8^+^ T cells are activated upon SARS-CoV-2 infection and *CXCR2* gene expression was significantly upregulated in severe and critical patients in the peripheral blood [64]. CCL2 was increased in the blood of infected individuals and elevated expression of the *CCR2* was associated with low counts of circulating inflammatory monocytes, indicating their recruitment into the inflamed lungs. Interestingly, ex vivo stimulation of peripheral blood mononuclear cells indicated reduced production of IFN-α in response to TLR stimuli from plasmacytoid DCs of SARS-CoV-2-infected patients compared with those of healthy control individuals [65]. Despite the above lack of type I IFN gene expression and protein production, upregulation of genes involved in type I IFN signaling was observed [60, 64, 65], and sensitive ELISA revealed a marked increase in blood IFN-α concentration peaking at 8 days after symptom onset [65]. Thus, low quantities of type I IFNs produced early in the lungs may circulate and cause a transient burst of type I IFN signaling in the peripheral blood. Of particular interest, the expression of TNFSF14 or LIGHT is distinctively elevated in the plasma of SARS-CoV-2-infected individuals [65]. As TNFSF14 is produced from activated T cells and acts on macrophages and fibroblasts to induce a number of proinflammatory and fibrogenic cytokines including IL-6, GM-CSF, and TGF-β [66, 67], it is plausible that T-cell production of TNFSF14 may induce both regeneration of alveolar structures and consolidation.

Although serum Ab responses induced upon SARS-CoV-2 infection are relatively short-lasting and subsided within a few months after symptom onset [44, 68], previous analyses of T-cell responses in SARS-CoV-infected individuals have shown that memory CD4^+^ and CD8^+^ T cells can be detected in recovered patients for as long as four years after infection (reviewed in [69]). Virus-specific CD4^+^ T cells detected at 2 years after infection in recovered individuals exhibited central memory while CD8^+^ T cells effector memory phenotypes, and CD8^+^ T cells produced high levels of IFN-γ and TNF-α upon peptide stimulation [70]. It should be noted, however, that as interstitial T cell infiltration is commonly observed in pathological specimens of COVID-19 pneumonia along with macrophage accumulation in alveolar lumina, TNF-α production from effector T cells may also contribute to damaging to alveolar walls and might further induce endothelial cell injury known as lymphocytic endotheliitis.

In mouse models of IAV infection, it has been shown that airway-resident CD8^+^ memory T cells are generated in specific niches associated with regenerative changes of consolidating lesions [71]. However, these CD8^+^ memory T-cell depots ate short-lived and conversion of peripherally circulating effector memory cells to airway-resident memory cells is inefficient [72]. Thus, if putative resident memory T cells detected in BALF of SARS-CoV-2-infected individuals are indeed associated with effective control of progression from moderate to serve/critical pneumonia, ways to direct differentiation of lung-resident CD8^+^ memory T cells might facilitate the development of both preventative and therapeutic measures for this respiratory infection currently causing an unprecedented global pandemic.

## Conclusions

In IAV infection, resident alveolar macrophages along with type I IFNs function to limit initial viral spread within infected lungs. However, the spatially excessive spread of IAV infection results in the vicious cycle of inflammatory monocyte and neutrophil infiltration through the feed-forward circuit of CCR2-mediated monocyte recruitment and CCL2 production from monocyte-derive macrophages. These inflammatory cells induce DAD through the expression of TNF-α, NOS, and TRAIL. Preexisting IAV-specific memory CD4^+^ T cells can control the spread of IAV from the upper to lower respiratory tract and can reduce morbidity. In SARS-CoV-2 infection, the virus can directly infect the lower respiratory tract and induce alveolar pathology often without severe systemic symptoms. Mechanisms of tissue injury in SARS-CoV-2-induced pneumonia seem to share some aspects with IAV-induced pneumonia in which monocytes and neutrophils play crucial roles. However, in the lungs of COVID-19 patients, interstitial lymphocyte infiltration is more pronounced along with the accumulation of macrophages in alveolar lumina, and reactivation of virus-specific memory CD8^+^ T cells may contribute to limit the progression of mild disease into severer and more critical lung pathologies. It should be noted that despite the possible importance of T-cell responses in protecting lungs from fatal damage, T cells might also be involved in the induction of endothelial cell injury [73], which is a rather unique characteristic of COVID-19 pathology. Further studies are required to dissect putative protective and possible pathogenic roles of T-cell responses in SARS-CoV-2 infection.

## Data Availability

All data generated or analyzed during this study are included in this published article.

## Abbreviations

Ab: Antibody
ACE: Angiotensin-converting enzyme
ARDS: Acute respiratory distress syndrome
BALF: Bronchoalveolar lavage fluid
CoV: Coronavirus
COVID-19: Coronavirus disease 2019
DAD: Diffuse alveolar damage
DC: Dendritic cell
IAV: Influenza A virus
IFN: interferon
MERS: Middle East Respiratory Syndrome
NOS: Nitric oxide synthase
NSP: Non-structural protein
ORF: Open reading frame
PID: Post-infection day
RTC: Replication and transcription complex
S: Spike
SARS: Severe Acute Respiratory Syndrome
TLR: Toll-like receptor
WT: Wild-type
